# Back to the sea twice: identifying candidate plant genes for molecular evolution to marine life

**DOI:** 10.1186/1471-2148-11-8

**Published:** 2011-01-12

**Authors:** Lothar Wissler, Francisco M Codoñer, Jenny Gu, Thorsten BH Reusch, Jeanine L Olsen, Gabriele Procaccini, Erich Bornberg-Bauer

**Affiliations:** 1 Evolutionary Bioinformatics, Institute for Evolution and Biodiversity, University of Muenster, Huefferstrasse 1, D48149 Muenster, Germany; 2 IRSI-Caixa Fundation, Hospital Universitari Germans Trias I Pujol, Crta de Canyet s/n 08916 Badalona, Spain; 3 Leibniz-Institut fuer Meereswissenschaften IFM-Geomar, Duesternbrooker Weg 20, D24105 Kiel, Germany; 4 Dept. Marine Benthic Ecology and Evolution, Centre for Ecological and Evolutionary Studies, University of Groningen, Centre for Life Sciences, Nijenborgh 7, 9747 AG Groningen, The Netherlands; 5 Stazione Zoologica A Dohrn, Villa Comunale, 80121 Naples, Italy

## Abstract

**Background:**

Seagrasses are a polyphyletic group of monocotyledonous angiosperms that have adapted to a completely submerged lifestyle in marine waters. Here, we exploit two collections of expressed sequence tags (ESTs) of two wide-spread and ecologically important seagrass species, the Mediterranean seagrass *Posidonia oceanica *(L.) Delile and the eelgrass *Zostera marina *L., which have independently evolved from aquatic ancestors. This replicated, yet independent evolutionary history facilitates the identification of traits that may have evolved in parallel and are possible instrumental candidates for adaptation to a marine habitat.

**Results:**

In our study, we provide the first quantitative perspective on molecular adaptations in two seagrass species. By constructing orthologous gene clusters shared between two seagrasses (*Z. marina *and *P. oceanica*) and eight distantly related terrestrial angiosperm species, 51 genes could be identified with detection of positive selection along the seagrass branches of the phylogenetic tree. Characterization of these positively selected genes using KEGG pathways and the Gene Ontology uncovered that these genes are mostly involved in translation, metabolism, and photosynthesis.

**Conclusions:**

These results provide first insights into which seagrass genes have diverged from their terrestrial counterparts via an initial aquatic stage characteristic of the order and to the derived fully-marine stage characteristic of seagrasses. We discuss how adaptive changes in these processes may have contributed to the evolution towards an aquatic and marine existence.

## Background

Lambers and co-authors summarized the uniqueness of seagrasses as follows: "Aquatic angiosperms are perhaps comparable to whales: They returned to the water, preserving some features of terrestrial organisms" [1]. The monocotyledonous seagrasses represent, in fact, a polyphyletic group of plants that can live underwater in fully marine environments. At least three independent seagrass lineages, but no other angiosperm species, have evolved to a life in the marine environment [2,3].

Seagrasses consist of about 60 species, most of which superficially resemble terrestrial grasses of the family Poaceae in that they have long, narrow leaves and grow in large meadows. Seagrasses belong to the order of Alismatales which includes 11 families of aquatic-freshwater species and 4 families that are fully marine. The marine families include the Posidoniaceae, Zosteraceae, Hydrocharitaceae, and Cymodoceaceae, and have originated in the Cretaceous period [2]. Phylogenetic analysis of members of the entire order, based on the plastid gene encoding for RuBisCO large subunit [4], indicates that the return into the sea occurred at least three times independently through parallel evolution from a common aquatic-freshwater ancestor of terrestrial origin.

Living submerged in an aqueous environment poses many challenges requiring physiological and morphological adaptations that are distinctive from terrestrial angiosperms. For example, the photosynthetic apparatus needs to be modulated to accommodate the changes in light attenuation through the water depth [5]. Consequently, the overall light intensity is decreased and the wavelength composition of sunlight reaching underwater plants is different. Accordingly, seagrasses have one of the highest light requirements among angiosperms [6,7]. One factor contributing to these high light requirements is the reducing sediments to which seagrasses are rooted. These sediments challenge seagrass root tissues with anaerobic conditions since marine sediments are often oxygen deficient. When the internal transport of oxygen from shoot to root tissues is not sufficient, seagrasses can be forced to resort to fermentative metabolism [8,9]. Submergence also exposes organisms to the forces of wave action and tidal currents that effects reproductive functions and reduces the availability of carbon dioxide (CO_2_). Consequently, seagrasses have evolved to propagate via hydrophilous pollination [10] and rely on carbonic acid and bicarbonate instead of CO_2 _[11,12]. Specific to marine environments, seagrasses are often exposed to high salt levels and short-term salinity fluctuations in the coastal and estuarine system [13-15]. Increased levels of sodium (Na^+^) are known to be toxic, partly due to the fact that both Na^+ ^and potassium (K^+^) have very similar physicochemical properties. Key metabolic processes in the cytoplasm such as enzymatic reactions, protein synthesis, and ribosome functions rely on K^+ ^as a co-factor [16]. An increased level of Na^+ ^creates a competing environment for K^+ ^binding sites and thus decreases efficiency of these processes. Moreover, detrimental effects can propagate from the cytoplasmic compartment into the chloroplasts, leading to a decreased efficiency of photosynthesis which in turn impairs growth [17].

Strikingly, despite their independent evolutionary routes, seagrasses from the three different lineages have evolved many similar morphologies, life history strategies, and breeding systems [3,18]. This indicates that the aquatic habitat imposes novel selection forces that can lead to parallel evolution. For instance, most seagrass species share a secondarily simplified morphology which includes horizontal rhizomes and strap-like leaves originating from a basal meristem. Additionally, seagrasses have been found to share morphological traits that distinguish them from terrestrial plants such as reduced stamen and corolla, and elongated pollen without exine walls [19]. Except for the genus *Enhalus *with above-surface pollination, all of the 60 seagrass species exhibit true sub-aqueous pollination by means of filiforme pollen (*hydrophily*; [10]). This adaptation to a marine habitat is thus an example of morphological parallel evolution [20,21].

Identifying genes and cellular processes that may have adaptive contributions to submerged fully marine habitats is therefore of particular interest. By comparing a group of marine angiosperms to terrestrial angiosperms, consequences of specific selection pressures and molecular adaptations can be uncovered. In general, such phenotypic changes can be caused by both changes in gene expression and the primary sequence of encoded proteins. Protein sequences can be strongly conserved whereas changes in their expression pattern can be adaptive (e.g. [22,23]). Conversely, changes in the coding sequence of genes can modify protein structure, function, and efficiency, and therefore can be used to identify evidence for parallel or convergent evolution as successfully demonstrated in recent studies for sequences in plants [24,25], monkeys [26], and fish [27,28].

In this study, the molecular evolution of an identified set of orthologous genes through changes in the coding sequences is investigated to identify candidate genes that may be involved in morphological and physiological adaptations of seagrasses. Gene expression changes as a second mechanism of phenotypic adaptation will not be addressed due to the limitation of the current dataset, although intra-specific analysis of EST libraries between heat-stressed and control treated *Zostera marina *has previously been conducted [29]. Comparing orthologous gene sequences of two seagrasses and eight terrestrial angiosperm species allows for the inference of sequence evolution and the statistical assessment of synonymous (*dS*) and non-synonymous (*dN*) substitution rates, providing insights into molecular adaptation [30,31] of seagrasses. We use EST libraries which were recently developed for two important seagrass species, the Mediterranean seagrass *Posidonia oceanica *(L.) Delile and the temperate species *Zostera marina *L. (eelgrass). These seagrass species are two representatives of three currently recognized independent seagrass lineages (Posidoniaceae and Zosteraceae) [4]. Using a molecular evolution approach, the positive selection (*dN/dS *> 1) of genes along branches leading to each seagrass species was investigated to identify candidate genes in which adaptations allowed for the transition from a terrestrial to an aquatic - and ultimately marine - lifestyle. Estimates of evolutionary distances can be obtained from the timetree database [32], which lists molecular sequence studies that determined that the two seagrass species *Z. marina *and *P. oceanica *split 72.5 to 75 million years ago [33-35] and their evolutionary distance to the terrestrial monocots used in this study is estimated at 131 million years [35].

## Results

### Construction of the dataset

The aim of this study was to investigate the molecular evolution of genes shared between seagrasses and terrestrial angiosperms following the split from the aquatic, last common ancestor (LCA) of seagrasses from the terrestrial monocots. In order to represent two independent seagrass lineages, expressed sequence tag (EST) data were used for *Zostera marina *and *Posidonia oceanica*. Orthologous sequences of the two seagrasses were compared to eight terrestrial angiosperm species with a balanced representation of monocot and dicot clades: four monocots including *Zea mays *[36], *Sorghum bicolor *[37], *Oryza sativa *[38] and *Brachypodium distachyon *[39]; and four dicots including *Arabidopsis thaliana *[40], *Populus trichocarpa *[41], *Medicago truncatula *[42], and *Vitis vinifera *[43]. Using sequences from all species, orthologous gene clusters (with one sequence per species) could be constructed for 189 genes. The genomes of the moss *Physcomitrella patens *[44] and green alga *Chlamydomonas reinhardtii *[45] were not included in this analysis as these species have split from higher plants roughly 600 and 900 million years ago, respectively. Evolutionary distances of this magnitude would have prevented accurate estimations of mutation rates.

### Detection of positive selection after the seagrass splits from terrestrial monocots

**Figure 1:**
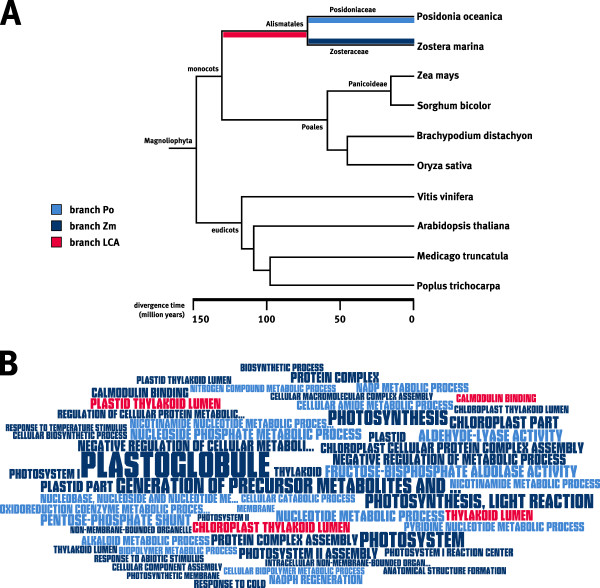
**Identification of positively selected genes in two seagrass species and their last common ancestor**. (A) Phylogenetic tree of ten plant species among which the molecular evolution of orthologous gene sequences has been analyzed. Positive selection in seagrass evolution has been tested for each of the three highlighted branches Po, Zm and LCA. Divergence times have been obtained from [34,76-80] and the timetree database [32]. (B) Term cloud of over-represented GeneOntology (GO) terms of positively selected genes compared to all tested genes. For each of the three tested branches, enriched GO terms were determined using all other tested genes as a reference as indicated by the different colors. The size of the GO terms is proportional to the *p*-value obtained in the enrichment test. This procedure creates a representation similar to sequence logos [81], showing enriched annotation terms instead of sequence conservation patterns. A tabular representation of the enriched GO terms can be found in Additional File 5.

Using a maximum likelihood framework, the sequence evolution of each gene was evaluated along the species tree (Figure 1A) by estimating the ratio (*ω*) between the rates of non-synonymous (*dN*) and synonymous substitutions (*dS*) in the coding sequence. The parameters used for the analysis were set such that only the alternative hypothesis allows for positive selection in the foreground branch, and a likelihood ratio test (LRT) can determine whether or not the alternative model is a significantly betterfit to the observed sequence alignment than the null model. To each orthologous gene cluster, the branch site test for positive selection in CODEML (test 2, [46]) was applied, using the evolutionary model that allows for a varying *ω *within the alignment and thus is sensitive towards positive selection limited to a very small number of sites. Testing for positive selection includes running CODEML twice, both with model A (model = 2; NSsites = 2) but with different constraints for the site classes (see Materials and Methods). Three branches abbreviated Po, Zm, and LCA (see Figure 1A) were used as foreground branches in separate tests to identify positive selection after the split of the two seagrass lineages from the terrestrial monocots. For each model, a likelihood score was obtained and a LRT was performed to test for positive selection with *p *< 0.05. Separate testing of the three branches allowed for rare cases where a gene was inferred to be positively selected in more than one branch. Accordingly, this approach uncovered 65 cases across 51 genes, where the branch-site test for positive selection was significant at least once for the three tested foreground branches (Table 1, *p *< 0.05).

**Table 1:** Genes with evidence for positive selection in seagrasses

Branch	Cluster ID	Arabidopsis gene description	***p*****-value**
LCA	orthomcl1184	60 S ribosomal protein L14 (RPL14A)	<0.001
LCA	orthomcl3768	proteasome maturation factor UMP1 family protein	0.001
LCA	orthomcl1461	annexin 7, calcium-dependent phospholipid binding (ANNAT7)	0.001
LCA	orthomcl1674	cytochrome c oxidase 6B (COX6B)	0.002
LCA	orthomcl538	chaperonin 20, calmodulin binding (CPN20)	0.003
LCA	orthomcl4618	PSAE-1	0.004
LCA	orthomcl5414	light harvesting complex PSII 5 (LHCB5)	0.004
LCA	orthomcl1707	ubiquinol-cytochrome C reductase subunit, mitochondrial	0.005
LCA	orthomcl3901	nuclear encoded CLP protease 5 (CLPP5)	0.006
LCA	orthomcl1048	ferredoxin 3 (ATFD3)	0.007
LCA	orthomcl4111	calmodulin binding (PSAN)	0.007
LCA	orthomcl1171	protease inhibitor/seed storage/lipid transfer protein (LTP)	0.007
LCA	orthomcl1074	60 S ribosomal protein L37 (RPL37A)	0.007
LCA	orthomcl433	lipid transfer protein 3, lipid binding (LTP3)	0.008
LCA	orthomcl1625	fructose-bisphosphate aldolase, putative	0.008
LCA	orthomcl3789	PHD finger protein-related	0.009
LCA	orthomcl801	60 S ribosomal protein L9 (RPL90B)	0.013
LCA	orthomcl1693	UDP-D-apiose/UDP-D-xylose synthase 1 (AXS1)	0.016
LCA	orthomcl1038	60 S ribosomal protein L18 (RPL18C)	0.020
LCA	orthomcl922	glutaredoxin 4, metal ion binding (GRX4)	0.021
LCA	orthomcl1070	60 S ribosomal protein L6 (RPL6B)	0.022
LCA	orthomcl2948	scorbate peroxidase 4 (APX4)	0.025
LCA	orthomcl1822	FK506 binding/peptidyl-prolyl cis-trans isomerase (FKBP15-2)	0.025
LCA	orthomcl626	40 S ribosomal protein S3A (RPS3aB)	0.025
LCA	orthomcl3845	copper ion bindng/electron carrier (DRT112)	0.026
LCA	orthomcl1565	40 S ribosomal protein S15 (RPS15C)	0.028
LCA	orthomcl4326	PQ-loop repeat family protein/transmembrane family protein	0.041
Po	orthomcl469	copper ion binding/glutamate-ammninoa ligase (ATGSR1)	<0.001
Po	orthomcl1126	cytidylate kinase/uridylate kinase (PYR6)	0.001
Po	orthomcl4752	glycine dehydrogenase, decarboxylating (GDCH)	0.002
Po	orthomcl1625	fructose-bisphosphate aldolase, putative	0.003
Po	orthomcl1125	40 S ribosomal protein S24 (RPS24B)	0.005
Po	orthomcl1070	60 S ribosomal protein L6 (RPL6B)	0.007
Po	orthomcl1673	cytochrome c-2 (CYTC-2)	0.008
Po	orthomcl1473	C2 domain-containing protein	0.011
Po	orthomcl1450	fructose-bisphosphate aldolase, putative	0.014
Po	orthomcl824	mitochondrial ATP synthase g subunit family protein	0.014
Po	orthomcl4197	enhancer of sos3-1, metal ion binding/protein binding (ENH1)	0.018
Po	orthomcl4326	PQ-loop repeat family protein/transmembrane family protein	0.022
Po	orthomcl1896	microsomal glutathione s-transferase, putative	0.028
Po	orthomcl5121	frostbite 1, NADH dehydrogenase, ubiquinone (FRO1)	0.028
Po	orthomcl2960	unknown protein	0.029
Po	orthomcl1930	cornichon family protein	0.041
Po	orthomcl433	lipid transfer protein 3, lipid binding (LTP3)	0.043
Po	orthomcl1635	histone H1-3 (HIS1-3)	0.046
Zm	orthomcl2446	photosystem I subunit L (PSAL)	0.001
Zm	orthomcl1812	PS II subunit O-2, oxygen-evolving/poly(U) binding (PSBO2)	0.003
Zm	orthomcl538	chaperonin 20, calmodulin binding (CPN20)	0.003
Zm	orthomcl3901	nuclear encoded CLP protease 5 (CLPP5)	0.004
Zm	orthomcl433	lipid transfer protein 3, lipid binding (LTP3)	0.005
Zm	orthomcl414	structural constituent of ribosome	0.005
Zm	orthomcl4111	calmodulin binding (PSAN)	0.007
Zm	orthomcl591	RuBisCO activator (RCA)	0.008
Zm	orthomcl1057	photosystem II subunit R (PSBR)	0.009
Zm	orthomcl953	dormancy-associated protein-like 1 (DYL1)	0.010
Zm	orthomcl3260	malate dehydrogenase, cytosolic, putative	0.012
Zm	orthomcl1450	fructose-bisphosphate aldolase, putative	0.014
Zm	orthomcl5948	prefoldin 6, unfolded protein binding (PDF6)	0.015
Zm	orthomcl1565	40 S ribosomal protein S15 (RPS15C)	0.017
Zm	orthomcl1808	universal stress protein (USP) family protein	0.031
Zm	orthomcl824	mitochondrial ATP synthase g subunit family protein	0.032
Zm	orthomcl4705	chlorophyll binding (LHCA3)	0.043
Zm	orthomcl3845	copper ion bindng/electron carrier (DRT112)	0.044
Zm	orthomcl4618	PSAE-1	0.045
Zm	orthomcl3789	PHD finger protein-related	0.049

Orthologous gene clusters with evidence for positive selection in at least one of the tested branches leading to *Zostera marina *(Zm), *Posidonia oceanica *(Po), and their last common ancestor (LCA; see Figure 1A). Each cluster was annotated using the TAIR9 functional description of the representative *A. thaliana *ortholog. *P *-values represent the significance of positive selection inferred by the branch-site test for positive selection.

### Annotation of positively selected genes (PSGs)

Among the 189 tested genes, 51 genes were identified as positively selected genes (PSGs). Using KEGG pathway information, 30 of the 51 PSGs could be associated to at least one pathway. Metabolic pathways, ribosomes, and photosynthesis showed the highest number of associated genes (Table 2), indicating that several components of these pathways have acquired sequence changes after the split of the common ancestor of seagrasses from the terrestrial monocots 130 MYA. For 27 of the 51 PSGs, positive selection was inferred in the branch leading to the last common ancestor of the two seagrass species (branch LCA, Figure 1A). Signals of positive selection in the LCA branch reflect either adaptation before the split of the two seagrass lineages or parallel evolution after their split. In the LCA branch, positive selection has been inferred mostly for ribosomal and metabolic genes (Table 2). Over-representation analysis of GO terms associated with PSGs in the LCA branch revealed only two functional gene classes significantly enriched, including proteins interacting with calmodulin, and proteins located in the thylakoid lumen (Figure 1B).

**Table 2:** KEGG pathways that are associated to PSGs

Map.ID	Map.Title	total	Po	Zm	LCA
01100	Metabolic pathways	16	6	8	9
03010	Ribosome	8	2	1	7
00195	Photosynthesis	7	0	6	4
00190	Oxidative phosphorylation	4	2	1	2
00710	Carbon fixation in photosynthetic organisms	3	2	2	1
01061	Biosynthesis of phenylpropanoids	3	2	2	1
01062	Biosynthesis of terpenoids and steroids	3	2	2	1
01063	Biosynthesis of alkaloids derived from shikimate pathway	3	2	2	1
01064	Biosynthesis of alkaloids derived from ornithine, lysine and nicotinic acid	3	2	2	1
01065	Biosynthesis of alkaloids derived from histidine and purine	3	2	2	1
01066	Biosynthesis of alkaloids derived from terpenoid and polyketide	3	2	2	1
01070	Biosynthesis of plant hormones	3	2	2	1
00010	Glycolysis/Gluconeogenesis	2	2	1	1
00030	Pentose phosphate pathway	2	2	1	1
00051	Fructose and mannose metabolism	2	2	1	1
00196	Photosynthesis - antenna proteins	2	0	1	1
00480	Glutathione metabolism	2	1	0	1
00020	Citrate cycle (TCA cycle)	1	0	1	0
00053	Ascorbate and aldarate metabolism	1	0	0	1
00240	Pyrimidine metabolism	1	1	0	0
00250	Alanine, aspartate and glutamate metabolism	1	1	0	0
00330	Arginine and proline metabolism	1	1	0	0
00520	Amino sugar and nucleotide sugar metabolism	1	0	0	1
00620	Pyruvate metabolism	1	0	1	0
00630	Glyoxylate and dicarboxylate metabolism	1	0	1	0
00910	Nitrogen metabolism	1	1	0	0
00980	Metabolism of xenobiotics by cytochrome P450	1	1	0	0
03050	Proteasome	1	0	0	1

For each pathway, described by the map ID and the title, the total number of associated PS genes are shown as well as the number of PSGs in each of the three branches Zm, Po, and LCA (see Figure 1A). Note that a gene can be associated to more than one pathway.

Potential lineage-specific positive selection was also detected for 18 and 20 PSGs in *Posidonia *and *Zostera*, respectively (Figure 1A, Table 2). Over-representation analysis using the Gene Ontology (GO) revealed that, within this limited sample, positive selection has acted on different functional classes between the three branches under investigation (Figure 1B). In the *Zostera lineage*, 6 PSGs were identified to be involved in the photosynthesis pathway (ID: 00195), whereas none of these were observed in the *Posidonia *lineage, suggesting that parts of the two photosystems and the light reaction have undergone *Zostera-*specfic adaptation (Table 2). Additionally, GO annotation indicates that in *Zostera*, positive selection has acted on genes responsive to abiotic stimuli and cold (Figure 1B). PSGs in *Posidonia *were identified to be mostly involved in metabolic processes and biosynthetic pathways. Together, these findings indicate that the two seagrass lineages have diverged substantially on a molecular level despite a seemingly similar habitat. Nonetheless, many signals of positive selection found in the LCA branch also indicate adaptive traits shared by both lineages. These PSGs may have evolved either in their last common ancestor or in parallel after their split.

## Discussion

### Positively selected genes associated with central biological pathways

Positive selection for 51 genes was detected after the split from terrestrial monocots based on a maximum likelihood approach. Theoretical models based on confirmed biological data have suggested that molecular adaptation is realized to different extents across the proteome and depends on the functional role of each individual protein [47]. In the present analysis, many of the identified PSGs are involved in the central biological pathways of translation, photosynthesis, and glycolysis. These adaptations are possibly associated to the above mentioned Na^+ ^toxicity which seagrasses have likely experienced during their evolution towards the marine environment. To this respect, molecular adaptation of key cellular processes known to be sensitive towards increased ionic levels such as photosynthesis, translation, and selected metabolic enzymes are expected. Considering the importance of these processes for the survival of an organism over short and evolutionary time scales, it is not surprising to identify strong selection pressure shaping genes which increase salt tolerance.

The available dataset allowed only for the investigation of 189 orthologous clusters, equivalent to ~1% of the *A. thaliana *genome. Since orthologous clusters include only ESTs from both seagrasses, the presented dataset is not an unbiased sample of the genome and is probably enriched for genes that show significant expression levels in both seagrass species. Nevertheless, the presented analysis was able to provide significant partial insights into the molecular evolution of seagrasses. While the limited size of the current dataset leaves room for further investigations, the well described ecology of seagrasses can be utilized to discuss how these PSGs may have contributed to seagrass adaptation to the marine environment.

### Molecular evolution for salt tolerance

A number of terrestrial lineages of plants have evolved into aquatic-freshwater hydrophytes and a number of morphological features are shared by both hydrophytes and seagrasses, e.g., the presence of a diffusive boundary layer around the leaves, a photosynthetic epidermis, loss of stomata and the development of aerenchyma (reviewed in [48]). Physiologically, however, seagrasses must cope with high ion concentrations, inefficient carbon uptake and other physical coping mechanisms that are still poorly understood. One of the questions that has to remain open is how exactly do seagrasses deal with the high salinity of the ocean. Seagrasses have been found to harbor increased intracellular levels of Na^+ ^and K^+ ^as compared to terrestrial angiosperm species [49] as well as to other aquatic angiosperms [48]. In general, salt-tolerant plants compensate osmotic and ionic imbalances with increased K^+ ^import and the accumulation of compatible solutes [50,51]. However, genes that are known to facilitate salt tolerance such as the SOS pathway [52,53] were absent from the orthologous gene clusters and could therefore not be investigated. Thus, the mechanism by which seagrasses achieve either a tolerance of higher salinity levels or employ active mechanisms to decrease intracellular Na^+ ^deserved further investigation with more comprehensive sequence and additional expression datasets.

### PSG Group 1: Glycolysis

With two fructose-bisphosphate aldolase enzymes and a malate dehydrogenase, the list of PSGs contains three enzymes of the glycolysis pathway. This observation may be particularly significant due to the challenges imposed by the O_2 _sink created by the reductive sediment leading to compensation by internal transport of oxygen from shoot to root tissues during the day cycle, as mentioned above. In darkness, seagrasses can even be forced to switch to fermentative metabolism. In *P. oceanica*, malate has previously been shown to accumulate as a consequence of anoxic conditions [54]. Hence, the positive selection of these three glycolysis genes may be associated with seagrass-specific adaptation to anaerobiosis.

### PSG Group 2: Ribosomal Genes

Ten PSGs were found to be ribosomal proteins involved in translation. From an evolutionary point of view, translation is an ancient cellular process, and high selection pressure is expected to act against deleterious mutations, as ribosome functioning affects virtually all cellular processes. In *A. thaliana*, on average four gene copies encode for any of the approximately 80 different ribosomal proteins [55,56]. This redundancy may reflect the importance of maintaining highly productive translation and protein synthesis. At least three scenarios can explain the seemingly high number of PSGs with ribosomal function: (1) Since translation is salt-sensitive, one can hypothesize that these changes reflect salt tolerance adaptations. The vast majority of signals of positive selection in ribosomes were inferred in the LCA branch so that these changes are shared by both seagrass species. Ultimately, signals of positive selection in ribosomes could be one of the traits that allowed the transition to the marine lifestyle. (2) As ribosomes consist of a multitude of subunits, changes in only a few proteins could cause compensatory mutations in other ribosomal proteins to maintain structure and function of the ribosomal complex. Such compensatory mutations were shown to occur in an *E. coli *mutant [57], and would increase the number of observed changes and overestimate the number of "adaptive changes". (3) Acquisition of non-ribosomal functions could explain sequence changes in these proteins without them being adaptive in the context of ribosomal functioning. In the primate ribosomal protein S4, positive selection has been shown to occur after gene duplication [58]. Andrés *et al*. [58] concluded that one gene copy has acquired a non-ribosomal function with 2 to 6 amino acid substitutions identified as positively selected sites. The three presented scenarios are not mutually exclusive and ultimately, more experiments will be required to reveal the nature of the inferred sequence changes.

### PSG Group 3: Photosynthesis and carbon fixation

Seven PSGs were related to the photosynthetic pathway and may reflect adaptations to new conditions of carbon fixation and photosynthesis that seagrasses had to face after their split from a terrestrial ancestor. Fixation of CO_2 _is expected to be more difficult for seagrasses since seawater contains very little dissolved carbon dioxide. While CO_2 _can readily diffuse from the air through the stomata to the mesophyll cells in terrestrial plants, aquatic plants often have limited CO_2 _diffusion rates [1]. Factors contributing to slow CO_2 _diffusion in aquatic plants (and especially in seagrasses) are thick boundary layers around the leaves that are sometimes amplified by the presence of unicellular or multicellular photosynthetic epiphytes that compete for CO_2 _[59], and the low rate of CO_2 _transport in water. The two seagrass species under investigation, *Z. marina *and *P. oceanica*, are known to utilize bicarbonate ($HCO_{3}^{-}$) as a major source of inorganic carbon for photosynthesis [11,12]. The ability to utilize $HCO_{3}^{-}$ could be one of the traits evolved in the LCA branch. In contrast, a set of signals of positive selection specific to the *Zostera *lineage could relate to the biochemical mechanism used in carbon fixation. Seagrasses have long been regarded as C3 plants, but physiological measurements have gathered indications that several seagrass species, including *Z. marina*, are C3-C4 intermediates or have various carbon-concentrating mechanisms to aid the RuBisCO enzyme in carbon acquisition [60-63]. Finally, seagrasses are able to activate different mechanisms to cope with conditions of light-limitation and shifted light spectrum [6,7] through long-lasting metabolic adjustments including down-regulation of RuBisCO, enhanced proteolysis [64] and putative changes in the antenna complex. These various unique characteristics of seagrasses are further supported by our results.

## Conclusions

We have undertaken the first step in systematically unraveling the molecular basis of seagrass evolution from terrestrial ancestors to a fully marine lifestyle. Only genes that were contained in the available seagrass EST collections could be analyzed in this study. Consequently, the current dataset of orthologous gene clusters for 10 angiosperm species is biased and limited in size. Nevertheless, this study has shed light on the molecular evolution of seagrass genes expressed under native conditions in root and leaf tissues. 51 genes showed evidence for positive selection in seagrass branches indicating that photosynthesis, a few metabolic pathways, and ribosomes have strongly diverged after the split of the common ancestor of seagrasses from terrestrial monocots. Further studies will need to address the following questions: (1) How seagrasses have acquired osmoregulatory capacity to tolerate high salinities, (2) how CO_2 _is fixated, (3) how their photosynthetic apparatus has evolved for under water light harvesting, and (4) under what conditions anaerobiosis takes place. In this regard, comparisons with the aquatic members of the Alismatales will be necessary to distinguish between more general adaptations to the aquatic environment and those that are marine-specific. Finally, the completion of the *Zostera marina *genome project, currently under way at the Joint Genome Institute (http://www.jgi.doe.gov/), will be a milestone in providing more comprehensive datasets in the near future to further our understanding of evolution and adaptation of seagrasses and their aquatic relatives.

## Methods

### Sequence data

Gene sequences from ten angiosperm species were compared to identify genes with signs of positive selection in seagrasses. The two seagrass species *Zostera marina *and *Posidonia oceanica *were represented by expressed sequence tag data. Protein-coding sequences from the genomes of eight terrestrial angiosperm species were used to contrast the seagrass sequences. These species included *Zea mays*, *Sorghum bicolor*, *Oryza sativa*, *Brachypodium distachyon*, *Arabidopsis thaliana*, *Populus trichocarpa*, *Medicago truncatula*, and *Vitis vinifera*. In the seagrass ESTs representing putative transcript sequences, open reading frames (ORFs) were predicted based on significant BLASTX matches [65] to protein sequences of the other eight angiosperm species (*E *< 1*e*^-5^). Two sequence datasets were constructed: one containing protein sequences, and another one for the protein coding sequences (CDS). For more information on the EST sequences and how the libraries were built can be found in [66].

### Orthologous gene clusters

Using the protein sequences of the ten species, orthologous gene clusters were constructed with OrthoMCL [67] using default settings. Only clusters with at least one sequence per species were used in our analysis. If more than one sequence of any species was contained in an OrthoMCL cluster, all sequences of that species were removed except for the one sequence that showed the highest similarity to all other sequences of the cluster as assessed with T-Coffee [68]. For each 1:1 ortholog cluster (see Additional file 1), multiple sequence alignments (MSAs) of the protein and coding sequences were constructed. First, protein sequences were aligned with MUSCLE [69] (see Additional file 2). Second, PAL2NAL [70] was applied to align the CDS codon-wise, guided by the protein MSA as a reference (see Additional file 3).

### Test for positive selection

CODEML from the PAML package [71] (v4.3) was used to identify genes under positive selection using a codon-based maximum likelihood method [72]. The phylogenetic relationships between the 10 tested taxa were obtained from NCBI Taxonomy (http://www.ncbi.nlm.nih.gov/Taxonomy/) and used as reference tree. To test a foreground branch for positive selection, CODEML was run twice, both with model A (model = 2; NSsites = 2) but with different constraints for the site classes as described for test 2 [46]. Model A requires branches in the tree to belong to either foreground or background branch category, where only foreground lineages are allowed to have experienced positive selection (*ω *> 0). Four classes of sites are assumed in model A: (1) class 0 codons are conserved through the tree, with 0 ¡ *ω*_0 _< 1; (2) class 1 codons evolve neutrally with *ω*_1 _= 1; (3) class 2a and (4) class 2b codons differ in their selection mode between foreground and background branches. In background branches, 2a codons are conserved with 0 <*ω *_0 _< 1, and 2b codons are neutral with *ω*_1 _= 1. In foreground branches and the null hypothesis run, 2a and 2b codons evolve neutrally with fixed *ω*_2 _= 1. In foreground branches under the alternative hypothesis, 2a and 2b codons are positively selected with each *ω*_2 _> 1. In separate runs, each of the three branches Zm, Po, and LCA were marked as foreground branches and the branch site test for positive selection was applied (see Additional file 4). Positive selection was inferred if the LRT between the scores of the models corresponding to the null and the alternative hypothesis was < 0.05. The *p*-values were not adjusted for multiple testing for two reasons. First, the presented dataset is relatively small, and given a 5% error rate, only about 3 false positives are to be expected among the 65 significant cases of positive selection. Second, lowering the *p*-value cutoff makes the test for positive selection a lot more conservative, dismissing genes where positive selection is limited to a very small number of residues.

### GeneOntology and KEGG pathway annotation

The *A. thaliana *ortholog of each cluster was used to associate additional annotation to the whole ortholog cluster. First, GeneOntology (GO) annotation [73] was obtained for *Arabidopsis thaliana *from http://www.geneontology.org/. Both filtered and unfiltered gene associations of *A. thaliana *(8 Dec 2009 version) were pooled. From these pooled annotations, only non-redundant mappings to genes from the newest Arabidopsis genome release (TAIR9) were kept. Based on the Arabidopsis ortholog contained in each cluster, GO terms were mapped to PSGs. The R package topGO [74] was used to test enrichment of GO annotation terms in these PSGs, using all tested orthologous clusters as reference (see Additional file 5). Enrichment was assessed by Fisher Exact tests as implemented in topGO's classic algorithm treating each GO term as an independent unit. Second, *A. thaliana *KEGG pathway annotation [75] was obtained from ftp://ftp.genome.jp/pub/kegg/genes/organisms/ath/, and mapped to the ortholog clusters via the Arabidopsis gene id.

## Authors' contributions

This study was conceived by TBHR and GP and designed by LW, EBB and FMC. LW performed all computations with help from FMC. Data were interpreted by LW, EBB and FMC. The paper was written by LW, JG, GP, JLO, and EBB, and corrected and approved by all authors.

## Supplementary Material

Additional file 1 **OrthoMCL cluster composition after clustering two seagrass EST datasets and 8 full angiosperm genomes**. Click here for file

Additional file 2 **Aligned proteins of each OrthoMCL cluster, produced with MUSCLE**. Click here for file

Additional file 3 **Aligned nucleotide (CDS) sequences of each OrthoMCL cluster, produced with PAL2NAL**. Click here for file

Additional file 4 **CODEML output of the branch-site test for positive selection for each of the three tested seagrass branches**. Click here for file

Additional file 5 **Results of the GeneOntology (GO) enrichment analysis, testing the PSGs of each branch against all genes tested for positive selection**. Click here for file
